# DISPARITIES IN DEPRESSION AMONG CHINESE OLDER ADULTS WITH NEURODEGENERATIVE DISEASES

**DOI:** 10.1093/geroni/igz038.3220

**Published:** 2019-11-08

**Authors:** Zhichao Hao, qingyi Li, Nicole Ruggiano

**Affiliations:** University of Alabama, Tuscaloosa, Alabama, United States

## Abstract

Depression is a major health issue among older adults, and it exerts negative impacts on them physically and mentally. In turn, various factors facilitate or impede the occurrence of depression, socially, economically and culturally. At the same time, neurodegenerative diseases have become a leading cause of death and disability worldwide. In China, the incidence rate of Parkinson’s disease among older adults aged 65 and older is 1.7%, which means 100,000 new cases occur each year, more than 2.5 million in total. Meanwhile, 3.21% of incidence rate, more than 8 million older adults aged 65 and older with Alzheimer’s Disease and Related Dementia (AD/RD) makes China become the largest and fastest-growing area of AD/RD in the world. Around 2050, Chinese older adults with AD/RD will exceed 20 million. However, little is known about the extent that to which older adults with Parkinson’s or AD/RD in China will suffer from depression. This study was conducted on the latest wave (2011-2014) of the Chinese Longitudinal Healthy Longevity Survey (CLHLS, 1998-2014). The sample included 334 Chinese older adults aged 65 and older with neurodegenerative diseases (Parkinson’s or AD/RD). A univariate and binomial hierarchical logistic regression were performed. Result showed that 13.5% (n = 45) participants reported depression. Several covariates were significantly correlated with the occurrence of depression, including: co-residence of interviewee, activity level, level of chronic diseases, self-reported health status and Instrumental Activity of Daily Life. Implications for research, policy, and practice are discussed.

